# Protocol for mapping tumor infiltration by murine T cells using multiplex immunofluorescence

**DOI:** 10.1016/j.xpro.2026.104570

**Published:** 2026-05-14

**Authors:** Raymond J. Lim, Camelia Dumitras, Kostyantyn Krysan, Linh M. Tran, Samantha Man, Steven M. Dubinett, Bin Liu

**Affiliations:** 1 Division of Pulmonary and Critical Care, Department of Medicine, David Geffen School of Medicine at University of California, Los Angeles, Los Angeles, CA 90095, USA; 2 Department of Molecular and Medical Pharmacology, David Geffen School of Medicine at University of California, Los Angeles, Los Angeles, CA 90095, USA; 3 Department of Medicine, VA Greater Los Angeles Healthcare System, Los Angeles, CA 90073, USA; 4 Department of Pathology and Laboratory Medicine, David Geffen School of Medicine at University of California, Los Angeles, Los Angeles, CA 90095, USA; 5 Jonsson Comprehensive Cancer Center, University of California, Los Angeles, Los Angeles, CA 90095, USA

**Keywords:** Cell Biology, Cancer, Immunology

## Abstract

Here, we present a 7-plex immunofluorescence staining protocol that enables the spatial analysis of murine tumor tissues to investigate the complex interplay between immune cells and the tumor microenvironment. We used a sequential antibody labeling strategy that identifies CD4^+^ and CD8^+^ T cells and characterizes their activation and regulatory states. This protocol also maps T cell infiltration within tumors, allowing researchers to investigate immune cell distribution, interactions, and the dynamic biology of the tumor immune landscape in murine tumor models.

For complete details on the use and execution of this protocol, please refer to Lim et al.^1^

## Highlights

• Optimized 7-plex immunofluorescence staining for murine T cell states in tumors
• Maps T cell infiltration and subset distribution to define immune contexture
• Provides detailed insight into T cell behavior at tumor borders and adjacent regions

## Abstract (editor highlights)

Publisher’s note: Undertaking any experimental protocol requires adherence to local institutional guidelines for laboratory safety and ethics.

## Graphical abstract

**Figure:**
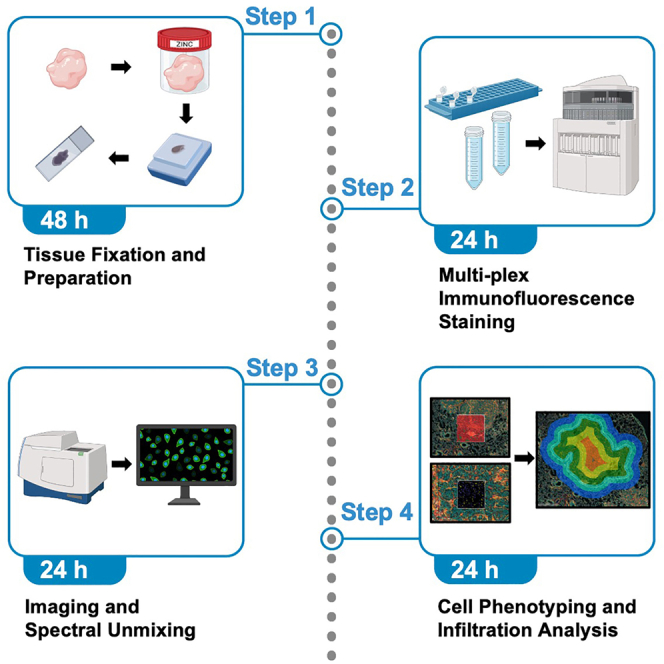

## Before you begin

Accurate spatial characterization of T cell subsets within tumors is essential for understanding antitumor immunity and therapeutic responsiveness. Distinct T cell states (i.e., cytotoxic effector T cells, regulatory T cells, and proliferating subsets) play divergent roles in tumor progression, immune evasion, and response to immunotherapies. Their spatial organization, density, and interactions with tumor and stromal cells can strongly influence outcomes in approaches, such as immune checkpoint blockade and other T cell–dependent treatments. Consequently, experimental workflows that preserve tissue context while enabling reliable discrimination among T cell functional states are increasingly important.

**Figure 1:**
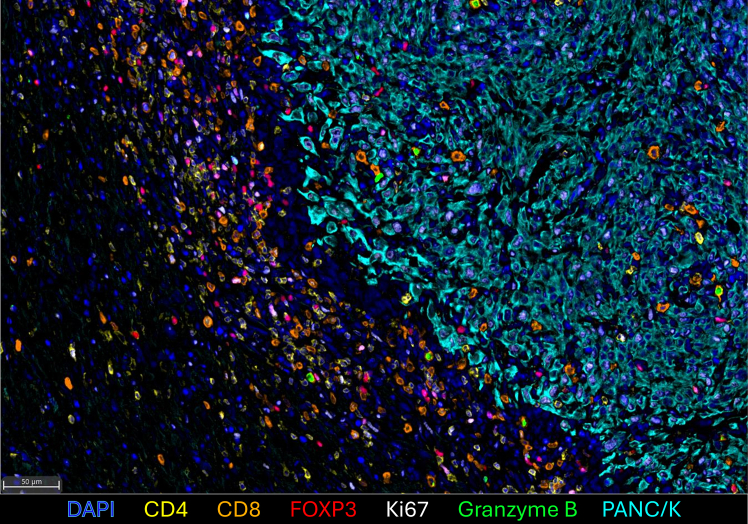
7-plex immunofluorescent staining of murine tumors highlighting T cell markers Scale bar = 50 μm.

This protocol provides a streamlined method for 7-plex immunofluorescent staining of murine tumor sections, enabling simultaneous visualization of multiple T cell markers alongside nuclear segmentation and functional readouts (Figure 1). When integrated with spatial analysis pipelines, the resulting datasets support the generation of quantitative maps of T cell distribution, infiltration patterns, and cell–cell interactions across the tumor microenvironment. This approach avoids the extensive panel development and analytical burden associated with many spatial omics platforms while still allowing efficient processing of multiple tumor regions to obtain a comprehensive view of T cell behavior. These spatial maps can reveal T cell–dense zones, the balance between effector and regulatory subsets, and proximity relationships with tumor or stromal compartments, providing insight into T cell activation, exhaustion, and suppressive states while complementing single-cell sequencing datasets by validating cell–cell relationships directly within intact tissue. Importantly, the spatially resolved datasets offer sufficient resolution to characterize immune organization in situ and to uncover emerging behaviors and interaction patterns of T cells in a scalable, high-throughput manner.

### Innovation

This protocol presents an optimized workflow for 7-plex immunofluorescent staining that enables high-resolution spatial quantification of T cell states within murine tumors. Compared with standard 3–4-plex panels, this method integrates a higher degree of multiplexing to reliably visualize cell-cell interactions and facilitating accurate spatial identification of T cell subsets and their functional states in the tumor microenvironment in a single tissue section. The panel design minimizes spectral overlap, enabling reliable co-detection of nuclear, cytoplasmic, and membrane markers within the same tissue section.

By integrating multiplexed staining with downstream spatial analysis pipelines, this protocol supports the generation of detailed maps of immune infiltration, effector activity, and regulatory T cell localization across the tumor microenvironment. This provides a more integrative and context-preserving view than traditional immunohistochemistry or flow cytometry, which lack spatial resolution. The ability to delineate T cell positioning and interaction patterns is especially valuable for applications such as CAR-T therapy,^2^ immune checkpoint blockade,^3^ and other T cell–based immunotherapies,^4^^,^^5^ where therapeutic efficacy depends on T cell engagement with tumor and stromal compartments.

### Institutional permissions

Samples were collected from tumors grown in mice. Mice were housed in pathogen-free facilities at UCLA, and all animal studies were approved by the UCLA Animal Research Committee (ARC protocol # 2017-049). All laboratory procedures are approved by the UCLA Institutional Biosafety Committee (BUA-2019-233-012-A).

### Tissue fixation

**Timing: 24–48 h**

1. Zinc Fixative Preparation.
  a. 10X Zinc Fixative (Formalin Free) – Dilute the 10x stock solution to 1x working concentration with MilliQ water.
    ***Note:*** Zinc fixation has been reported in the literature to provide better signal recovery from CD4 and CD8 markers.^6^ Paraformaldehyde (PFA) or formalin fixation can be used as an alternative; however, staining performance can vary, and verification of staining is recommended when using fixatives.
  b. Fix tissues by placing freshly dissected tissue pieces <3mm in size in Zinc Fixative for 24–48 h at 20–25^o^C.
  c. Following fixation, rinse tissue with deionized water and store in 70% Ethanol before embedding in paraffin blocks. When ready to stain, section the tissue at 5 μm thickness using a microtome and mount them onto slides.

### Preparation for Discovery Ultra reagents and antibodies

**Timing: 3 h**

2. DISCOVERY Wash – Dilute the 10x stock solution to 1x working concentration with MilliQ water and load into the reservoir.
3. Reaction Buffer Concentrate (10X) – Dilute the 10x stock solution to 1x working concentration with MilliQ water and load into the reservoir.
4. Liquid Cover Slip (LCS) – Load LCS to the reservoir.
5. Benchmark Ultra CC1 – Load CC1 to the reservoir.
6. Benchmark Ultra CC2 – Load CC2 to the reservoir.
7. Option Reservoir- Load Reaction Buffer (not used).
8. DISCOVERY Inhibitor (DISC Inhibitor)- Ready to Use Reagent Dispenser.
9. Pretreatment 1 Dispenser – 50 mM Copper Sulfate dissolved in MilliQ water.

***Note:*** Copper may not be required for formalin fixed tissue.

10. Antibody 1 Dispenser– Granzyme B (Diluted 1:100 in Antibody Diluent).

***Note:*** All antibody and reagent preparations listed here refer to the concentrations loaded into the automated dispenser. The final working concentration on the slide is further diluted upon mixing with reaction buffer (100 μL dispenser volume + 300 μL buffer; 1:4 dilution), resulting in a correspondingly on-slide working concentration (e.g., 1:100 becomes 1:400).

11. Antibody 2 Dispenser– FOXP3 (Diluted 1:100 in Antibody Diluent).
12. Antibody 3 Dispenser– CD8 (Diluted 1:100 in Antibody Diluent).
13. Antibody 4 Dispenser– CD4 (Diluted 1:200 in Antibody Diluent).
14. PanCK – Ready to Use Reagent Dispenser.
15. Antibody 5 Dispenser – Ki67 (Diluted 1:100 in Antibody Diluent).
16. Detection 1 Dispenser– Opal 480 (Diluted 1:100 in 1X Amplification Diluent).
17. Detection 2 Dispenser– Opal 520 (Diluted 1:100 in 1X Amplification Diluent).
18. Detection 3 Dispenser– Opal 570 (Diluted 1:100 in 1X Amplification Diluent).
19. Detection 4 Dispenser– Opal 620 (Diluted 1:100 in 1X Amplification Diluent).
20. Detection 5 Dispenser– Opal 690 (Diluted 1:100 in 1X Amplification Diluent).
21. Detection 6 Dispenser– TSA DIG (Diluted 1:100 in 1X Amplicfication Diluent).
22. Detection 7 Dispenser – Opal 780 (Diluted 1:12.5 in Antibody Diluent).
23. OmniMAP Anti-Rat HRP – Ready to Use Reagent Dispenser.
24. OmniMAP Anti-Rabbit HRP – Ready to Use Reagent Dispenser.
25. OmniMAP Anti-Mouse HRP – Ready to Use Reagent Dispenser.
26. Counterstain 1 Dispenser– Akoya DAPI (3 Drops in 1mL of MillliQ Water).

## Key resources table

**Table:** 

REAGENT or RESOURCE	SOURCE	IDENTIFIER
**Antibodies**		

Granzyme B Clone E5V2 L (1:400)	Cell Signaling Technology	Cat #44153; RRID:AB_2857976
FOXP3 Clone D6O8R (1:400)	Cell Signaling Technology	Cat #12653; RRID:AB_2797979
CD8 Clone 4SM15 (1:400)	eBiosciences	Cat #14-0808-82; RRID:AB_2572861
CD4 Clone 4SM95 (1:800)	eBiosciences	Cat #14-9766-82; RRID:AB_2573008
Ki67 Polyclonal Antibody (1:400)	Bethyl Laboratories	Cat #IHC-00375; RRID:AB_1547959
PanCK Clone AE1/AE3/PCK26 (Ready to use dispenser)	Roche	Cat #760-2135; RRID:AB_2810237
OmniMap Anti-Rat HRP (Ready to use dispenser)	Roche	Cat #760-4457; RRID:AB_3095527
OmniMap Anti-Mouse HRP (Ready to use dispenser)	Roche	Cat #760-4310; RRID:AB_2885182
OmniMap Anti-Rabbit HRP (Ready to use dispenser)	Roche	Cat #760-4311; RRID:AB_2811043

**Chemicals, peptides, and recombinant proteins**		

Copper Sulfate	Millipore Sigma	Cat #451657
Zinc fixative (10X)	BD Pharmingen	Cat #552658
Dawn Dish Soap	Office Depot/OfficeMax	Cat #172777

**Critical commercial assays**		

Benchmark ULTRA CC1	Roche	Cat #950-224
Benchmark ULTRA CC2	Roche	Cat #950-223
Reaction Buffer Concentrate (10×)	Roche	Cat #950-300
Liquid Cover Slip	Roche	Cat #650-210
DISCOVERY Wash	Roche	Cat #950-510
DISCOVERY Inhibitor	Roche	Cat #760-4840
Antibody Dispenser Kit	Roche	Cat #770-001
Detection Dispenser Kit	Roche	Cat #960-911
Pretreatment Dispenser Kit	Roche	Cat #960-901
Counterstain Dispenser Kit	Roche	Cat #771-741
Opal 480 (1:400)	Akoya Biosciences	Cat #FP1500001KT
Opal 520 (1:400)	Akoya Biosciences	Cat #FP1487001KT
Opal 570 (1:400)	Akoya Biosciences	Cat #FP1488001KT
Opal 620 (1:400)	Akoya Biosciences	Cat #FP1495001KT
Opal 690 (1:400)	Akoya Biosciences	Cat #FP1497001KT
Opal 780 (1:50)	Akoya Biosciences	Cat #FP1501001KT
Spectral DAPI	Akoya Biosciences	Cat #FP1490
Antibody Diluent	Akoya Biosciences	Cat #ARD1001EA
1× Plus Amplification Diluent	Akoya Biosciences	Cat #FP1498
ProLong Diamond Antifade Mountant	Molecular Probes	Cat #P36970

**Software and algorithms**		

inForm	Akoya Biosciences	https://www.akoyabio.com/phenoimager/software/inform-tissue-finder/
HALO	Indica Labs	https://indicalab.com/halo/
PhenoChart Whole Slide Viewer	Akoya Biosciences	https://www.akoyabio.com/support/software/

**Other**		

Ventana Discovery Ultra autostainer	Roche	https://diagnostics.roche.com/us/en/products/product-category/immunohistochemistry--ihc-/discovery-ultra.html
Vectra Polaris Imaging System	Akoya Biosciences	https://www.akoyabio.com/wp-content/uploads/2021/11/Vectra_Polaris_Product_Note_with_MOTiF_Akoya.pdf

## Materials and equipment

### Protocol creation on discovery ultra instrument

Use the RUO Discovery Universal procedure to create the staining protocol, following the steps listed below.

### Slide labels for discovery ultra instrument

Generate unique barcoded labels for sample slides which include names of sample and unique identifiers.

## Step-by-step method details

### Ventana Discovery Ultra automated slide staining

**Timing: 20 h**

This section details the step-by-step procedure for 7-plex immunofluorescence staining of Zinc-fixed murine tissue using the Ventana Discovery Ultra automated slide stainer. The step-by-step assay settings are programed into the instrument before the run.

1. Slide Deparaffinization:
  a. Bake slides for 32 min at 69°C.
  b. Deparaffin slides with DISCOVERY Wash for 32 min at 69°C.

***Note:*** As an alternative to the automated protocol, slides can be deparaffinized and rehydrated using a standard xylene-based protocol followed by graded ethanol washes and a final rinse in water.^6^

2. Antigen Retrieval and Background Quenching:
  a. Incubate the slide with Cell Conditioner 1 (CC1, Tris based buffer, pH 7.0–9.2) for 32 min at 95°C.
  b. Incubate with Copper Sulfate for 1 h at 37°C.
  c. Incubate with DISC Inhibitor for 16 min at 37°C.

***Note:*** As an alternative to automated processing, antigen retrieval may be performed using a standard water bath–based incubation of slides at 95 °C, maintaining stable temperature conditions, followed by cooling in the retrieval buffer and rinsing in Milli-Q water and TBST. Incubation for background quenching can also be performed with a standard water bath.^7^

3. Antibody Staining and Tyramide Curing/Antibody Stripping:
  ***Note:*** The staining order, antibody incubation conditions, antigen retrieval strategy, and fluorophore assignments described here were experimentally optimized to achieve balanced signal intensity, specificity, and minimal background. Fluorophores were assigned based on antigen abundance, and staining sequence was adjusted to reduce signal interference and carryover across cycles. These conditions represent the optimized protocol used in this study; detailed rationale, optimization considerations, and potential pitfalls are provided in the troubleshooting section.
  a. Incubate with primary antibody at 37°C. See Table 1 for recommended antibody conditions.

**Table 1:** Antibody conditions

Primary antibody	Primary incubation time	Secondary antibody	Secondary incubation time	Tyramide	Tyramide incubation time
Granzyme B	32 min	Anti-Rabbit HRP	16 min	Opal 520	16 min
FOXP3	32 min	Anti-Rabbit HRP	16 min	Opal 690	16 min
CD8	32 min	Anti-Rat HRP	16 min	Opal 620	16 min
CD4	32 min	Anti-Rat HRP	16 min	Opal 570	16 min
PanCK	32 min	Anti-Mouse HRP	16 min	Opal 480	16 min
Ki67	16 min	Anti-Rabbit HRP	16 min	Opal TSA-DIG	16 min
  b. Incubate with secondary antibody for 16 min at 37°C.
  c. Incubate with corresponding tyramide for 16 min at 37°C.
  d. Incubate in Cell Conditioner 2 for 8 min at 95°C.
4. Repeat Step 3 for each primary antibody.
5. Staining of Opal 780 Tyramide (Binding to Opal TSA-DIG).
  a. Following final incubation in Cell Conditioner 2, Incubate in Opal 780 for 1 h at 37°C.
6. DAPI counterstain and Coverslip.
  **CRITICAL:** Do not allow the tissue on the slide to dry out at any step in the process. Following washing, the slides can be stored briefly in DI water before adding mountant (Step 6b and 6c).
  a. Incubate slide in DAPI for 10 min at RT.
  b. Remove slide from Ventana Autostainer and remove LCS by thoroughly washing in 5% Dawn dish soap and rinsing with water.
  c. Apply Prolong Gold Antifade Mountant (50-100μL per slide).
  d. Apply coverslip and harden for at least 1 hour at 20–25^o^C, protected from light.
  e. Store at 4°C for long term storage. Keep protected from light.

### Slide imaging and analysis

**Timing: 48 h**

The following workflow outlines whole-slide image acquisition, spectral unmixing, cell segmentation, phenotype classification, and spatial infiltration analysis of multiplex-stained murine tumor sections. These steps enable single-cell quantification of T cell subsets and define their distribution across tumor regions.

7. Acquire whole-slide images using the Vectra Polaris or an equivalent platform capable of capturing multispectral fluorescence imaging compatible with Opals (≥7 channels).
8. Use Phenochart (or equivalent viewer such as QuPath) to review slides, select representative regions of interest (ROIs), and batch-export fields for downstream analysis.
9. Perform spectral unmixing using inForm to separate overlapping fluorophores and remove autofluorescence based on a prebuilt spectral library.

***Note:*** Linear unmixing can also be performed in QuPath when spectral reference images are available.

**Figure 2:**
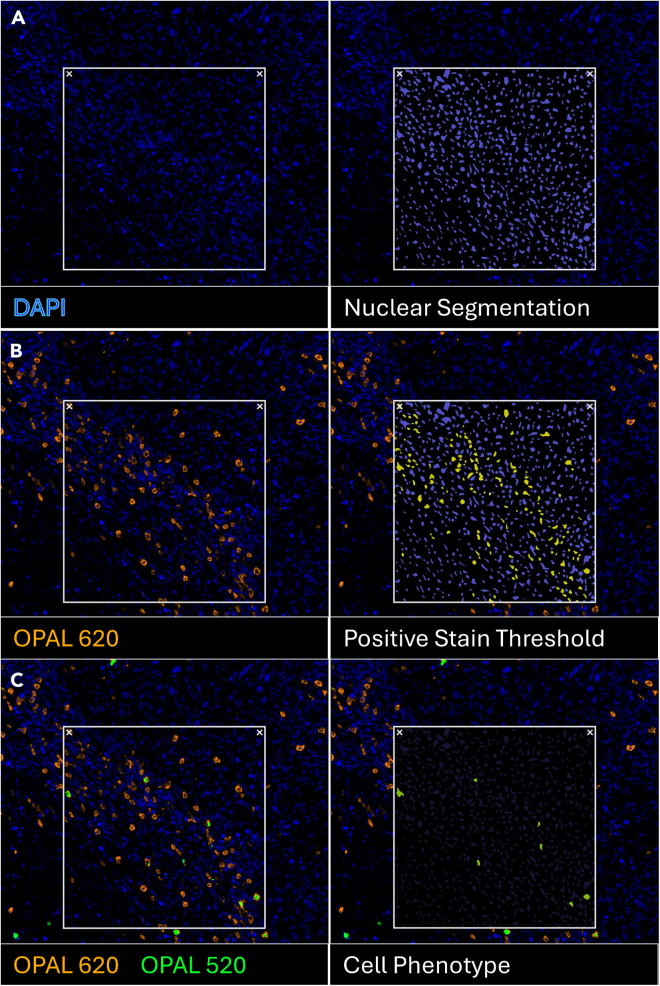
Analysis workflow for cell phenotyping (A) nuclear segmentation for identification of individual cells; (B) intensity thresholding to determine Opal stain positivity; (C) biological assignment of cell phenotypes.

10. Whole-slide assembly by stitching unmixed image tiles into a full-resolution whole-slide image can be done using HALO or QuPath if needed.
11. Use the Halo Tissue Classifier Module to generate classifiers to differentiate the glass slide from tissue in the image (can also be done in QuPath).
12. Perform nuclear detection and cell segmentation using HALO (Inform, QuPath or Fiji ImageJ) to generate single-cell objects using the DAPI nuclear stains with optional cytoplasmic expansion (Figure 2A).

**Table 2:** Nuclear detection parameters

Nuclear Contrast Threshold	0.5
Minimum Nuclear Intensity	0.1
Maximum Image Brightness	0.1
Nuclear Segmentation Aggressiveness	0.7
Fill Nuclear Holes	False
Nuclear Size	1, 482.3009
Minimum Nuclear Roundness	0
Number of Nuclear Dyes	1
Nuclear Dye 1	Dapi
Nuclear D1 Weight	1

***Note:*** Nuclear detection was configured by adjusting the DAPI intensity threshold, background contrast, segmentation aggressiveness to separate clustered cells into single nuclei, and by defining expected nuclear size and shape. Based on the approach described above, Table 2 shows the current parameters used for nuclear detection and cell segmentation in Halo.

13. Define intensity thresholds for each Opal channel using HALO (alternatively Inform, QuPath or Fiji ImageJ) to classify as marker-positive or -negative.

***Note:*** Thresholds should be optimized using control regions and applied consistently across samples (Figure 2B). For example, our Halo parameters for Opal 570 (CD4 marker) were set at a positive threshold between 1.5 and 11. Nucleus % Completeness was set at 0.

14. In HALO, assign cell phenotypes by characterizing cells as shown in Table 3 based on positive or negative Opal staining (can also be performed with Inform, QuPath or Fiji ImageJ).

**Table 3:** Characterization of cell phenotypes

Cell phenotype	Opal					
	480	520	570	620	690	780
CD4^+^ T Cell	−	–	+	−	–	–
CD8^+^ T Cell	−	–	−	+	–	–
PanCK (tumor cell)	+	–	−	−	–	–
CD4^+^ FOXP3^+^	−	–	+	−	+	–
CD8^+^ Granzyme B^+^	−	+	−	+	–	–
CD4^+^ Ki67^+^	−	–	+	−	–	+
CD8^+^ Ki67^+^	−	–	−	+	–	+
PanCK^+^ Ki67^+^	+	–	−	−	–	+

***Note:*** This step converts multiplex intensity data into biologically interpretable populations (Figure 2C).

15. Using HALO, perform whole-slide quantification to assess differences in cell population composition across the tissue section.

***Note:*** Equivalent measurements can be generated in QuPath or exported as a single-cell spatial data CSV file for downstream analysis in R or Python.

16. For additional spatial infiltration analysis, define tumor regions in HALO based on PanCK staining to generate a tumor border annotation.

***Note:*** Annotation tools are available in QuPath or custom scripts in R or Python applied to exported CSV data.

**Figure 3:**
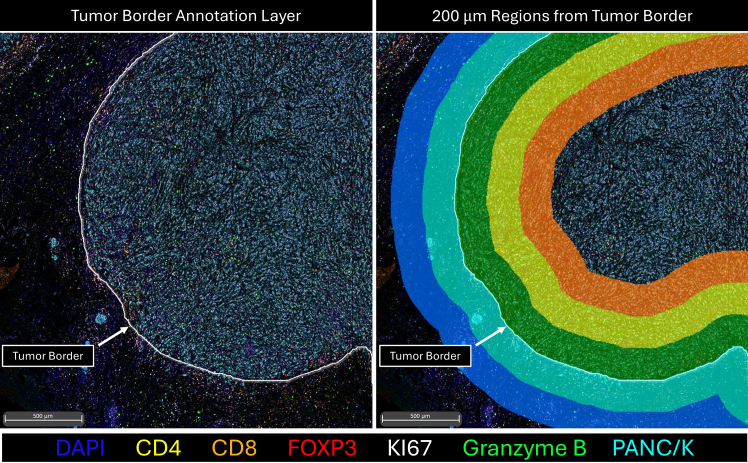
Tumor border annotation and generation of concentric band regions emanating from the tumor border Scale bars = 500 μm.

***Note:*** Equivalent analyses can also be performed in QuPath or via custom scripts in R or Python.

17. In the HALO Spatial Analysis Module, perform infiltration analysis by generating 200 μm regions inside (intratumoral) and outside (peritumoral) the tumor border for each cell type (Figure 3).

## Expected outcomes

**Figure 4:**
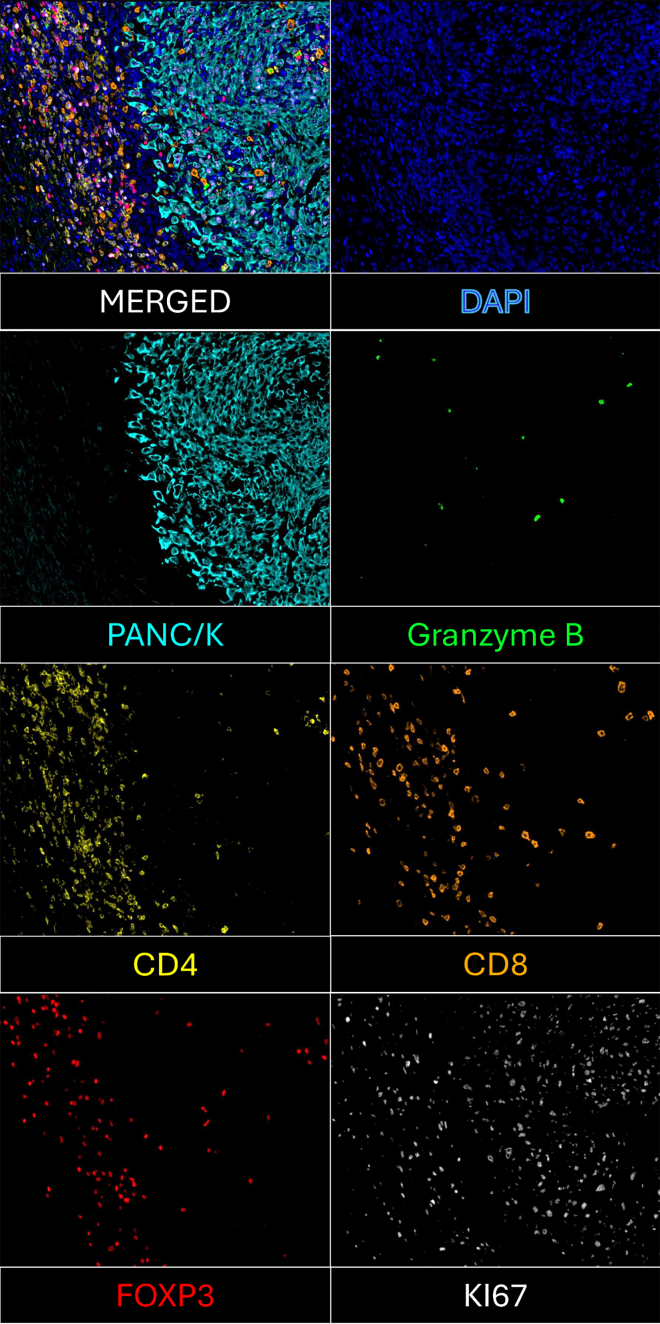
Representative images showing individual fluorescence channels and the corresponding merged composite

Successful completion of this 7-plex immunofluorescent staining protocol will yield high-resolution, multiplexed tissue images that distinctly resolve major T cell subsets within the tumor microenvironment (Figure 4). Researchers should first confirm robust DAPI staining to identify individual nuclei and ensure accurate cell segmentation during downstream analysis, which is essential for reliable classification of T cell populations.

**Figure 5:**
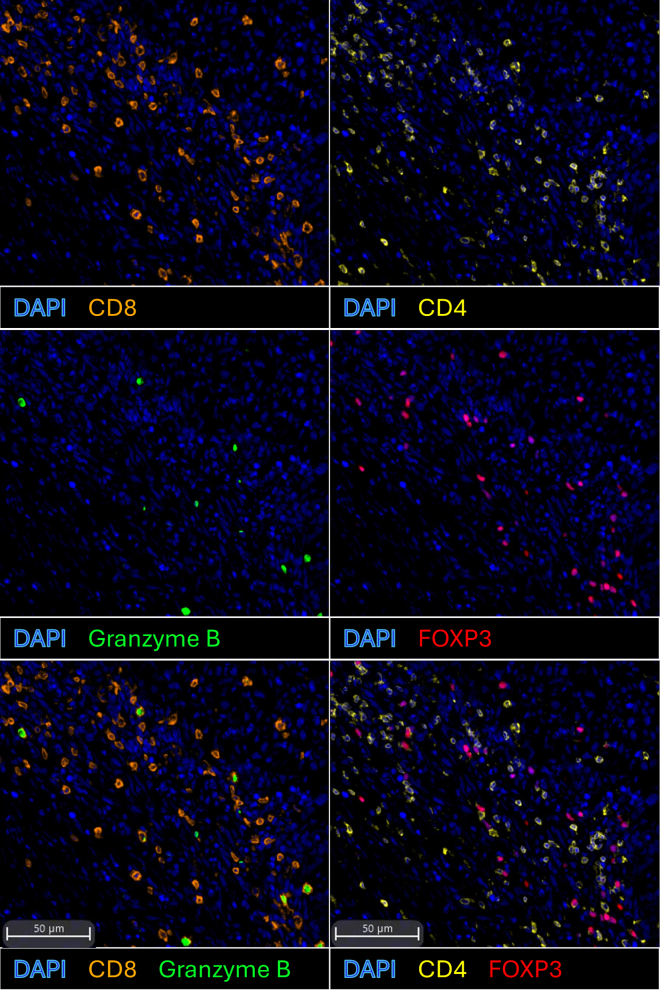
Co-localization of CD4^+^FOXP3^+^ and CD8^+^Granzyme B^+^ cells Scale bars = 50 μm.

Markers for CD4^+^ and CD8^+^ T cells are expected to show strong and specific membrane-associated signal with minimal spectral bleed-through. FOXP3 signal should appear as discrete nuclear staining within a subset of CD4^+^ cells, confirming the presence of regulatory T cells. Granzyme B should display cytoplasmic punctate or diffused staining within activated CD8^+^ effector cells. These spatial relationships, such as CD4^+^FOXP3^+^ and CD8^+^Granzyme B^+^ co-localization, serve as internal validation of correct panel performance. Representative results are shown in Figure 5. The protocol should also identify Ki67^+^ proliferating lymphocytes, which may be present in both CD4^+^ and CD8^+^ populations. When spectral unmixing and segmentation are optimized, researchers can expect clearly separated expression profiles for all seven markers at the single-cell level (see troubleshooting for common artifacts and optimization strategies).

Spatial analysis of the resulting datasets will typically reveal distinct T cell–dense regions within or around tumor nests and differential localization of effector (CD8^+^Granzyme B^+^) versus regulatory (CD4^+^FOXP3^+^) T cell subsets. This will allow for quantifiable proximity relationships between T cells and tumor or stromal compartments. These outcomes enable the construction of spatial phenotyping maps that quantify T cell densities across the tumor microenvironment, reflecting the overall magnitude of immune presence and activation. They also characterize the spatial infiltration patterns of T cell subsets at the tumor border, revealing whether immune cells are excluded from or penetrate tumor regions, and thereby distinguishing immune-excluded from inflamed tumor phenotypes. This also sets the foundation for further analysis that can be performed such as neighborhood analyses, and functional state distributions.^6^ The resulting datasets are sufficiently detailed to compare immune architecture across experimental conditions or treatment groups.

Overall, the protocol produces multiplexed images and spatially resolved data that complement flow cytometry and single-cell sequencing by preserving tissue context, allowing for the visualization of cell–cell interactions and microenvironmental organization in situ.

## Limitations

This protocol is optimized for execution on a Ventana Autostainer, and its reliability depends heavily on the controlled reagent delivery, incubation timing, and temperature regulation provided by that platform. When adapted for fully manual staining, users should expect increased variability in fixation sensitivity, antigen retrieval efficiency, and multiplex signal balance. Manual implementation introduces additional environmental and mechanical sources of variability, including fluctuations in incubation temperature, inconsistent reagent application, and differences in handling between operators. These factors may require substantial optimization of fixation conditions (troubleshooting problem 1), retrieval times (troubleshooting problem 3), and antibody concentrations (troubleshooting problem 3, 4, and 5) beyond those described here. Accordingly, while the current protocol offers a robust starting point, its performance outside the Ventana system will likely require iterative adjustment to achieve comparable staining quality and reproducibility.

## Troubleshooting

### Problem 1 (tissue fixation: Step 1)

Suboptimal fixation leading to weak staining and poor tissue integrity.

Uneven or weak staining within a tissue section, or inconsistent signal intensity across the same channel, is indicative of suboptimal fixation. This may also present as reduced reproducibility between samples. Because fixation efficiency varies with tissue size and composition, inconsistent signal across a section often reflects incomplete fixative penetration. Insufficient crosslinking can impair antigen preservation, reduce signal intensity, and compromise DAPI labeling whereas over-fixation may limit epitope accessibility. Consistent and complete fixation is required for reproducible multiplex immunofluorescence.

### Potential solution

• Fixative volume: Ensure tissue pieces remain fully submerged in an adequate volume of fixative to allow uniform penetration during the incubation.
• Tissue size: Trim tissue into smaller, evenly sized pieces to improve fixative diffusion and reduce regional variability.
• Fixation duration: Extend fixation at 4 °C for dense or slow-to-penetrate tissues, while avoiding prolonged incubation that may reduce antigen accessibility.
• Fixation selection: Zinc fixation may improve staining of certain immune markers (e.g., CD4, CD8), whereas formalin fixation can be used as an alternative but may require shorter fixation times and independent optimization depending on tissue type.^6^

### Problem 2 (Ventana staining step 2b and 2c)

High background fluorescence or non-specific signal.

Background signal may vary substantially between tissue types due to differences in intrinsic autofluorescence or sensitivity to amplification reagents. This may present as diffuse signal, reduced contrast, or false-positive staining. Elevated autofluorescence is often associated with insufficient discovery inhibitor or copper treatment, while excessive amplification can further increase non-specific signal.

### Potential solution

• Background inhibition steps: Optimize discovery inhibitor and/or copper incubation time to balance signal amplification with background suppression. Re-optimization of copper treatment may be required depending on tissue type and baseline autofluorescence.

***Note:*** Formalin-fixed tissue typically does not require copper treatment.

• Antibody optimization: Validate secondary antibodies to ensure minimal non-specific binding, as off-target interactions may contribute to background signal and be misinterpreted as autofluorescence.

### Problem 3 (Ventana staining step 2a and 3)

Weak or poor antibody staining for target epitopes.

Weak or inconsistent staining of specific markers may result from incompatibility between antigen retrieval conditions and antibody sensitivity. This may present as epitope-specific signal loss or absence of expected positive staining. Because antigen retrieval directly regulates epitope accessibility, suboptimal conditions can significantly impair antibody binding and downstream signal detection. Tissue type and fixation method influence response to CC1 and CC2 buffers; under-retrieval can result in weak signal, whereas over-retrieval may cause epitope damage or tissue degradation.

### Potential solution

• Antibody validation: Validate each antibody in singleplex under defined retrieval conditions prior to multiplexing to confirm expected staining patterns.
• Retrieval selection: Evaluate CC1 versus CC2 for each antibody. CC1 (basic, more stringent) improves recovery of heavily crosslinked epitopes, whereas CC2 (citrate-based, milder) is better suited for sensitive targets.
• Retrieval duration: Optimize retrieval time to balance epitope exposure and tissue preservation. Under-retrieval leads to weak signal, while over-retrieval may increase background or damage epitopes.
• Antibody incubation optimization: Adjust incubation time to balance signal intensity, specificity, and background. On the Ventana Autostainer, slides are maintained at 37 °C, enabling shorter incubations (e.g., 32 min primary and 16 min secondary). Manual staining may require longer incubation at 20–25^o^C or for 16-20 hours at 4 °C.
• Antibody titration: Perform titration for each primary antibody to determine the optimal concentration that maximizes signal while minimizing nonspecific staining.

### Problem 4 (Ventana staining step 3c)

Improper matching of Opal fluorophore intensity to antigen abundance.

Weak signal, oversaturation, or poor dynamic range may result from inappropriate pairing of fluorophore brightness with target antigen abundance. This can obscure biologically meaningful differences and limit interpretation of multiplex data.

### Potential solution

• Fluorophore assignment: Match fluorophore brightness to antigen abundance. Pair low-abundance targets with brighter fluorophores and highly expressed markers with dimmer fluorophores to prevent saturation.
  ○ Panel-specific optimization: In this panel, low-abundance markers (e.g., Granzyme B, FOXP3) were paired with brighter fluorophores (Opal 520, Opal 690), while more abundant markers (e.g., CD4, CD8, Ki67) were assigned to dimmer fluorophores (Opal 570, Opal 620, Opal 780).
  ○ Signal balancing: Reassign fluorophores if markers appear oversaturated or underrepresented relative to others.
• Tyramide optimization: Assess signal intensity of each channel to ensure adequate separation and dynamic range from neighboring channels. Signal intensity can be adjusted by modifying tyramide concentration or incubation time if needed.

### Problem 5 (Ventana staining step 3)

Signal loss or interference from one marker during multiplex staining.

Markers that perform well in single plex may exhibit reduced signal, increased background, or altered staining patterns when combined into a multiplex panel. This may result from cumulative antigen retrieval cycles, steric hindrance or fluorophore interference.

### Potential solution

• Single plex validation: Confirm that each antibody produces the expected staining pattern independently before multiplexing.
• Cycle simulation: Evaluate antibody performance under sequential retrieval conditions (e.g., repeated CC2 cycles) to assess signal stability when placed later in the staining order.
• Staining order optimization.
  ○ Antibody and Opal pairing: Low-abundance antigens were paired with brighter fluorophores to maximize signal detection, whereas highly expressed antigens were paired with comparatively dimmer fluorophores to prevent signal oversaturation.
  ○ Epitope abundance: Place low-abundance or retrieval-sensitive targets earlier in the sequence and assign robust or highly expressed markers to later cycles to prevent steric hindrance.
  ○ Marker separation: Separate markers with known co-expression (e.g., CD8 and Granzyme B) within the staining order to minimize false-positive co-localization due to signal carryover or bleed-through.
  ○ Fluorophore spacing: Avoid placing spectrally adjacent fluorophores (e.g., Opal 620 and Opal 690) in sequential staining cycles to identify potential signal overlap and interference.
• Retrieval flexibility: The current strategy uses CC1 initially, followed by CC2 (pH 6, 95 °C, 8 min). If an epitope is sensitive to CC1, an initial CC2 step can be used prior to CC1. Conversely, additional CC1 steps can be introduced later in the sequence for difficult epitopes, with careful validation to avoid disrupting prior signals.
• Panel iteration: Multiplex optimization is iterative. Adjust antibody concentration, staining order, fluorophore pairing, and retrieval conditions to achieve consistent performance across all targets.

***Note:*** Our combination supports epitope preservation and consistent signal across this staining panel with minimal changes required, however, changing antibodies may require further optimization of the entire panel.

### Problem 6 (slide imaging and analysis step 12)

Inadequate cell segmentation.

Failure to accurately segment individual cells is often associated with weak or uneven DAPI staining. This may present as merged nuclei, missed cells, or inclusion of background as false positives. Because DAPI is required for nuclear identification, insufficient signal intensity or contrast can compromise downstream cell classification. Suboptimal fixation or excessive antigen retrieval may further disrupt nuclear morphology, contributing to segmentation errors.

### Potential solution

• Fixation quality: If DAPI staining is weak or inconsistent, reassess fixation conditions (see Problem 1), as under-fixed tissue reduces nuclear integrity and DAPI signal.
• DAPI optimization: Adjust DAPI concentration or incubation time to improve nuclear signal intensity and contrast relative to background.
• Segmentation parameter optimization:
  ○ Adjust DAPI intensity thresholds to distinguish nuclei from background.
  ○ Modify segmentation aggressiveness to separate closely adjacent nuclei.
  ○ Use a broad nuclear size range to capture variability between small lymphocytes and larger tumor cells while excluding debris.
  ○ Avoid overly restrictive shape filters, as immune cells may exhibit irregular nuclear morphology.
• Augmented cell identification: If DAPI-based segmentation is insufficient, incorporate lineage markers (e.g., CD4, CD8) to aid identification of immune populations with weak nuclear staining.

## Resource availability

### Lead contact

Further information and requests should be directed to and will be fulfilled by the lead contact, Dr. Bin Liu (bliu@mednet.ucla.edu).

### Technical contact

For technical specifics on executing the protocol, Dr. Raymond J. Lim (raymondjohnlim@g.ucla.edu) and Camelia Dumitras (cdumitras@mednet.ucla.edu) will provide support to ensure its correct implementation.

### Materials availability

This study did not generate new reagents and materials.

### Data and code availability

All analyses were performed on HALO Software. Additional information and analysis parameters are available upon request.

## Acknowledgments

We thank Lauren Winter and Elvira Liclican for administrative support and editorial assistance. Schematic images were generated using BioRender. This work was supported in part by the 10.13039/100005188Tobacco-Related Disease Research Program (10.13039/100005188TRDRP) predoctoral fellowship award (T30DT0963 to R.J.L.), the UCLA Technology Development Group Innovation Fund (to S.M.D.), and the Merit Review Research Funds from the 10.13039/100000738Department of Veterans Affairs (1I01BX006419 to S.M.D.).

## Author contributions

R.J.L., C.D., and B.L. developed the study concept. R.J.L. and C.D. designed the experiments. R.J.L., C.D., and S.M. conducted the experiments. R.J.L., C.D., L.M.T., and K.K. performed the data analysis. S.M.D. and B.L. directed the study. R.J.L., C.D., and B.L. wrote the manuscript. All of the authors reviewed, revised, and approved the final manuscript.

## Declaration of interests

S.M.D. is a scientific advisory board member for EarlyDiagnostics Inc. and LungLife AI; he has received research funding from Johnson & Johnson and Novartis.
